# Estimative of real number of infections by COVID-19 in Brazil and possible scenarios

**DOI:** 10.1016/j.idm.2020.09.004

**Published:** 2020-09-24

**Authors:** H.P.C. Cintra, F.N. Fontinele

**Affiliations:** Physics Institute, University of Brasilia, Brasilia, Brazil

**Keywords:** COVID-19, SEIRD, Undernotification, Brazil

## Abstract

This paper attempts to provide methods to estimate the real scenario of the novel coronavirus pandemic in Brazil, specifically in the states of Sao Paulo, Pernambuco, Espirito Santo, Amazonas and the Federal District. By the use of a SEIRD mathematical model with age division, we predict the infection and death curves, stating the peak date for Brazil and above states. We also carry out a prediction for the ICU demand in these states and for how severe possible collapse in the local health system would be. Finally, we establish some future scenarios including the relaxation on social isolation and the introduction of vaccines and other efficient therapeutic treatments against the virus.

## 1 Introduction

In December 2019, the city of Wuhan in mainland China started experiencing an outbreak of unknown pneumonia cases. Later, the cause of this outbreak was identified as a virus belonging to the *Orthocoronavidae* subfamiliy and the *Betacoronavirus* genus (Cui et al., 2019), similar to the SARS-CoV virus that caused the SARS crisis in 2003 (Andersen et al., 2020). That similarity suggested the name SARS-CoV-2 to the novel coronavirus, and COVID-19 to the disease (Coronavirus Disease - 2019).

The virus quickly spread to other countries, reaching several countries by the end of February and being declared as a pandemic by the World Health Organization (WHO) on the 11th of March, being classified as a high risk threat for the world’s population (WHO, 2020). Since then, several mathematical models were used to predict the dynamics of the pandemic crisis in other countries. One of those models with the biggest impact was developed by Imperial College London (Ferguson et al., 2020).

In Brazil, the first case registered dates back to February 25th, but in this study we suggest evidence that the infection might have started 19–24 days before the official record. We then proceed to simulate the crisis in specific states and attempt to estimate the real scale of the outbreak, predicting when the infections peak might occur as well as the curve for ICU demand. Finally, we present some future scenarios based on how halting the intervention might affect the curve. We also explore how the introduction of vaccines or available medication might change the infection curve since there are several studies being made to evaluate the possible use of pharmaceutical drugs to cure the disease (McCreary & Pogue, 2020), (Negri et al., 2020) and (Tang et al., 2020).

## 2 Description of the model

We make use of a SEIRD model, dividing the population into 5 groups: Susceptible, Exposed, Infected, Recovered and Dead. The exposed population differs from the infected population in the development of their symptoms; an individual with the virus first enters first the exposed group, carrying the virus during its incubation period; then, after the incubation period, the individual passes to the infected group. The rate of infection is proportional to the number of infected and a contact constant β, which is given by the average number of contacts between individuals multiplied by the probability of contracting the virus during each contact. The development rate of symptoms is proportional to the incubation period $c^{-1}$. The rate of recovery γ is proportional to the percentage of people who recover divided by the average time taken from the onset of symptoms to recovery, similarly to the death rate μ. Another consideration is that people in the exposed group might infect susceptible people with an infection rate *k* which is a small fraction of β, that is, $k=P_{exp}\beta$, where $P_{exp}$ determines the fraction of infections caused by exposed individuals.

**Fig. 1:**
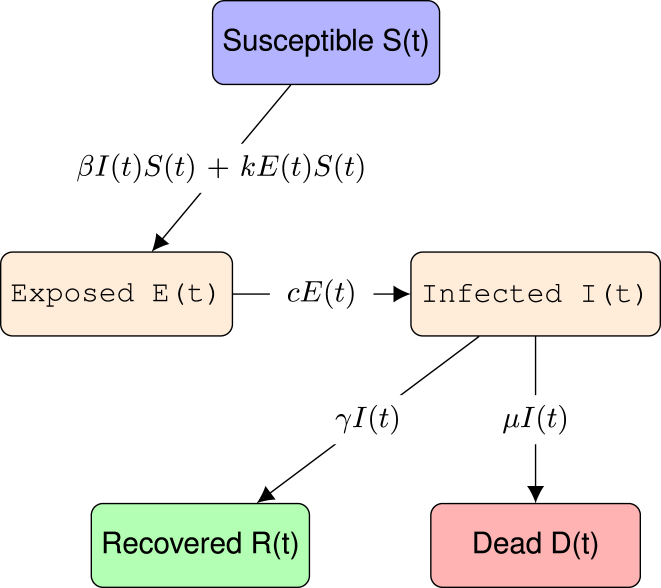
Representation of a SEIRD model, a susceptible person gets exposed to the virus, being infected afterwards and either dies or recovers from the disease.

The following diagram represents the dynamics of these populations (Fig. 1):

This model is represented by the following set of differential equations

(1) $$\frac{dS}{dt}=-\frac{\beta }{N}I\left(t\right)S\left(t\right)-\frac{k}{N}E\left(t\right)S\left(t\right)$$

(2) $$\frac{dE}{dt}=\frac{\beta }{N}I\left(t\right)S\left(t\right)+\frac{k}{N}E\left(t\right)S\left(t\right)-cE\left(t\right)$$

(3) $$\frac{dI}{dt}=cE\left(t\right)-\gamma I\left(t\right)-\mu I\left(t\right)$$

(4) $$\frac{dR}{dt}=\gamma I\left(t\right)$$

(5) $$\frac{dD}{dt}=\mu I\left(t\right)$$

where the recovery rate γ and death rate μ are represented in terms of the Infection Fatality Rate (IFR) $P_{IFR}$ and the average time from the onset of symptoms to recovery $\tau_{r}$ and death $\tau_{d}$.

(6) $$\mu =\frac{P_{IFR}}{\tau_{d}}$$

(7) $$\gamma =\frac{1-P_{IFR}}{\tau_{r}}$$

All equations described conserve the total population *N*, which is assumed constant and homogeneous for the model to be valid. This, of course, presents a limitation of the model, since in reality *N* is not homogeneous. Therefore, here *N* has the role of the effective population, being equivalent to the population which the virus might reach during an interval of some months. Estimating the real *N* is not an easy task, and in next sections we discuss how we decided to estimate this number.

We then divided the population into age groups to better describe how these rates vary from group to group. With that, we made the following changes to the model in accordance to what was proposed by (Rocha Filho et al., 2020):

(8) $$P_{IFR}\to P_{IFR_{i}},i=1,2,\cdots ,M$$

(9) $$\beta \to \beta_{i}=\overset{M}{\underset{j=1}{\sum }}P_{Inf}C_{ij}$$

where *M* is the number of age groups, $C_{ij}$ is the social contact matrix, representing the average contacts between a member of the *i*-th group with all other *j*-th groups and $P_{Inf}$ is the probability of being infected at each contact.

With these definitions, we represent non-pharmaceutical interventions such as social isolation and lockdown with a decrease of β given by a logistic function of the type

(10) $$\beta =\frac{P_{d}\beta_{i}}{1+\tau e^{t-t_{c}}}+\left(1-P_{d}\right)\beta_{i}$$

here, $\beta_{i}$ is the infection rate before the intervention, $t_{c}$ is the time when the intervention starts, $P_{d}$ is the fraction of reduction in infection rate achieved and τ is a constant related to the time taken from the start of the intervention until $P_{d}$ is reached.

When simulating the curve for infections and deaths in Brazil and in the states of Pernambuco, Espirito Santo, Sao Paulo, Amazonas and the Federal District, we used the model described above. Meanwhile, when simulating the ICU demand, we do not apply the age division for lack of specific data for each age group, thus, we apply the simple SEIRD model with β extracted from the fitting of data of each state and $P_{IFR}$, $\tau_{r}$ and $\tau_{d}$ appropriate for COVID-19 patients in the ICU.

## 3 Estimating the percentage of lost cases

### 3.1 Number of hospitalizations by SARS

According to (Salje et al., 2020), 3.6% of COVID-19 infections are severe and require hospitalization of which 30% are critical and require an ICU unit. Some studies found a hospitalization rate of around 14% (Wu & McGoogan, 2020). Yet another study found similar percentages, stating that 19% of the infections resulted in hospitalizations (COVID & Team, 2020). However, these studies calculate these fractions according to the registered cases, which are undernotified in many regions.

**Fig. 2:**
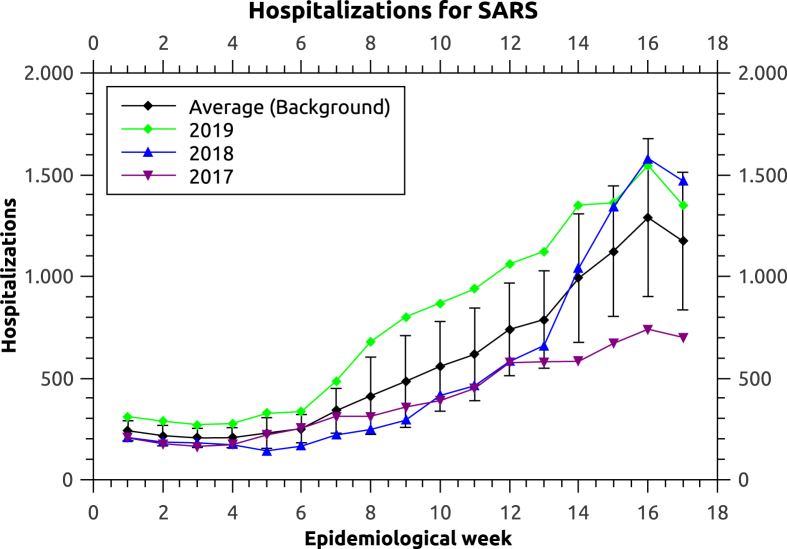
Hospitalizations by SARS in Brazil in the years of 2017, 2018 and 2019. The 18 weeks correspond to the period of January 01, 2017 to May 06, 2017 for 2017 (green), December 31, 2017 to May 05, 2018 for 2018 (blue) and December 30, 2018 to May 04, 2019 for 2019 (purple).

**Fig. 3:**
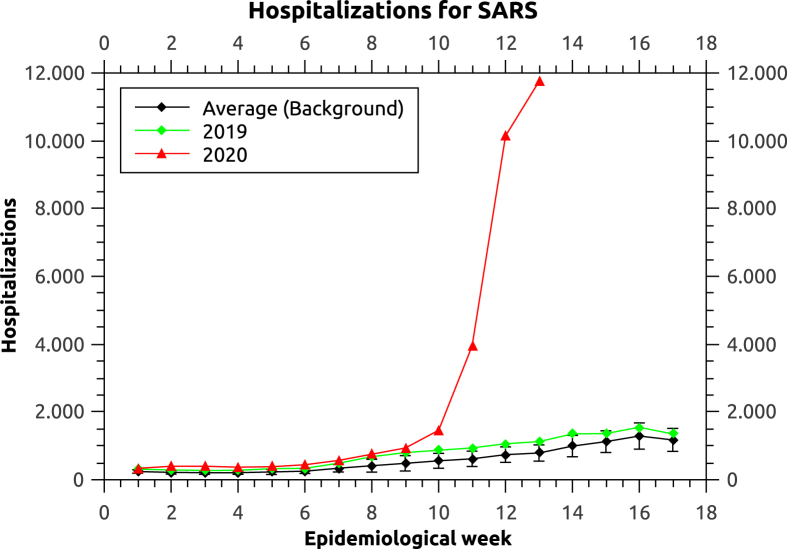
Number of hospitalizations by SARS in Brazil in the year of 2020, 2019 and the background average.

With the emergence of the novel coronavirus, the number of hospitalizations by SARS per week increased when compared to the years of 2019, 2018 and 2017. It is important to state that SARS hospitalizations here should not be misunderstood as caused by the SARS-CoV virus, responsible for the SARS epidemic in 2002 (Marra et al., 2003). In Brazil, the term SARS is also used to describe severe acute respiratory infection, independent of the etiological agent. Using the number of hospitalizations by SARS during these years, we build a value for the background behavior, that is, the expected number of hospitalizations due to other respiratory diseases (Fig. 2). The number reported by the Health Ministry per week is subject to alterations due to the fact that new results in the following weeks may be related to previous ones, as new results are released. For example, by the end of the 6th week of the year 2018, the official report estimates a number of hospitalizations of around 50 people, but later on, this number was corrected to be close to 200. Because of this uncertainty in the most recent data, we use the values available from four weeks before the most recent report (Fig. 3).

Fig. 3 shows an increase in hospitalizations by SARS in Brazil. Due to the definitions of SARS infection used by Brazils Health Ministry, most cases of hospitalizations by COVID-19 are diagnosed as SARS. Therefore, we assume that the large increase of SARS hospitalizations in comparison to the background is mainly caused by COVID-19. When comparing the years of 2018 and 2019, the latter presented an average increase (during the epidemiological weeks considered) of 10% in hospitalizations by other causes (Influenza, etc). We assume the same increase could be found from 2019 to 2020, thus, we consider that COVID-19 is responsible for 90% of the increase.

We observed that by the 6th week of 2020, the number of hospitalizations by SARS was 121 hospitalizations higher, compared to the upper error bar of the background value, and higher even than the year of 2019 by 106 hospitalizations, representing an increase of 31%, evidencing the likely existence of COVID-19 hospitalizations. According to a study performed on COVID-19 patients in Shanghai, the hospitalization occurs on average 4 days after the symptoms onset, ranging from 2 to 7 days (Chen et al., 2020). The study, together with the increase of SARS hospitalizations by the 6th week of 2020 suggests the possible existence of COVID-19 cases in Brazil between February 1st and February 6th, 19–24 days before the official record of the first case on the 25th of February.

Following the increase of hospitalizations, by the end of the 13th epidemiological week of 2020 (March 28, 2020), the number of hospitalizations by SARS in Brazil was already, 12260, while the background’s upper error bar of reaches a value of merely 1028, and only 1123 were registered in the year of 2019. From our assumption, 90% of the excessive hospitalizations are attributed to COVID-19, resulting in 10023–10108 hospitalizations by infections of the SARS-CoV-2 virus, which translates into 278416 to 280777 infections between March 21, 2020 and March 26, 2020 (According to the average time taken to be hospitalized). Comparing these estimates with the official numbers reported trough this period, we find a real number of infections 90 to 200 times bigger than the official number (125 times, using the average). That represents a loss of 99.2% (99.0–99.5) of actual infections. By comparison, a study done in China found that 86% infections were undocumented infections prior to 23rd january (Li et al., 2020a).

### 3.2 Number of tests performed

**Fig. 4:**
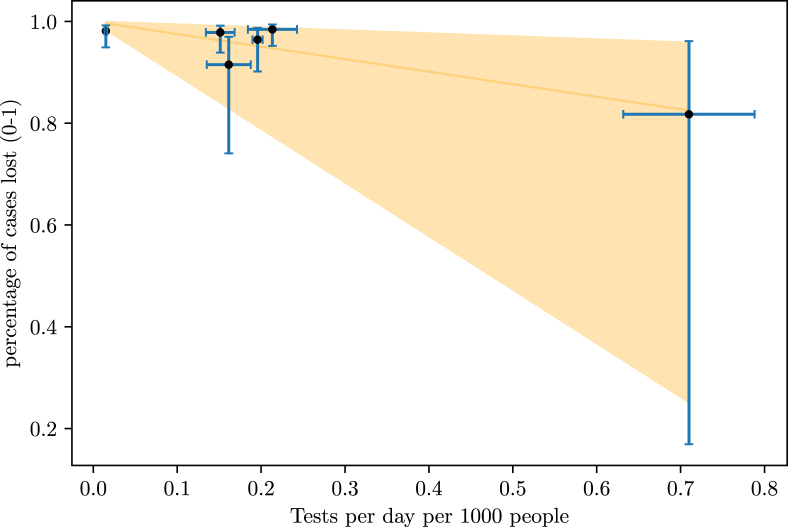
Relationship between the number of tests performed per day per 1000 inhabitants and the fraction of lost cases.

**Fig. 5:**
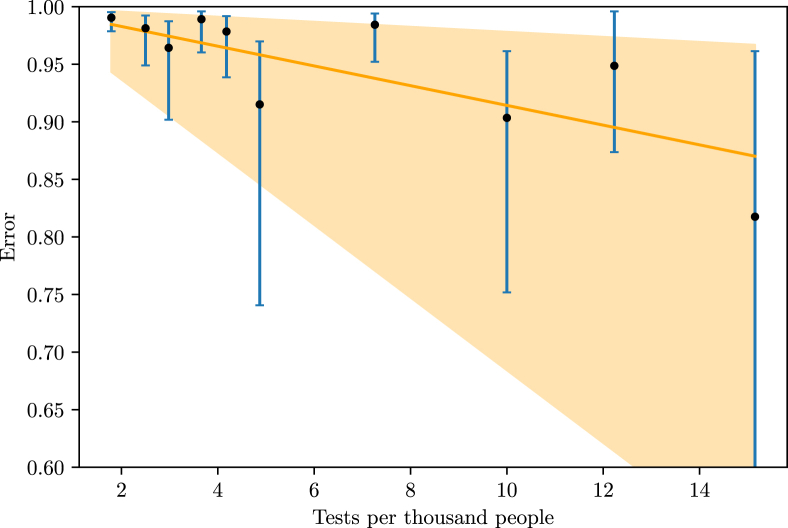
Relationship between the number of tests performed in total per 1000 inhabitants and the fraction of lost cases.

A study of Imperial College London estimated the number of infections in 11 European countries until March 28th, based on the basic reproduction number of the disease, found to be between 2 and 3 (Zhang et al., 2020), (Zhao et al., 2020), (Liu et al., 2020) and (Read et al., 2020), and the type of non-pharmaceutical intervention done by the countries on specific dates (Flaxman et al., 2020). With these estimations, we may find the percentage of lost cases, that is, infections not documented, in these countries until the 28th of March by comparing the estimated number of people infected with the official data available on the 28th of March. Comparing these percentages with the number of tests done per 1000 inhabitants and the number of tests done per day per 1000 inhabitants, we found a linear relation between the number of total tests done per 1000 inhabitants and the number of tests performed per day per 1000 inhabitants in a country, as well as and the percentage of lost cases (Fig. 4, Fig. 5).

The number of points on each graph is different because, although the study considered 11 countries, not all of them had data of tests per day available at (Max Roser & Ortiz-Ospina, 2020). The correlation between the number of tests per day with the fraction of undocumented infections is −0.90, and the correlation between the number of total tests with the fraction of undocumented infections is −0.79. A F-Test applied to the data set of both relations rejected the null hypothesis and that the variation of tests and the variation of undernotification are not significantly correlated (p < 0.05). We also compared the undocumented cases with the progression of the outbreak in each country and the day on which the non-pharmaceutical interventions were imposed, but found no correlation. We evaluated the effect of the increasing rate of testing as well, but it had no observable effect. From this comparison, a country needs to perform 4 (0.94–17 tests) tests per day per 1000 inhabitants in order to obtain a excellent track of infections. Here, the large margin for the higher values of testing arises from the low density of data points on the bigger values of the *x*-axis in Fig. 4.

The last official registration of the total number of tests done per 1000 inhabitants in Brazil of was 3.46, which corresponds to 97% of cases being lost (89–99.2%). However, the highest value released on the closest date to the 13th epidemiological week, reports 1.37 tests per 1000 inhabitants, corresponding to 98.8% of infections being undocumented (95.6–99.7%). A more precise number could be achieved with the data of tests per day per 1000 inhabitants, allowing a 2-dimensional regression. Unfortunately, we found no record of this information. Still, when fitting the data to a 2-dimensional regression algorithm, the resulting function states that the most important factor controlling the uncertainty of cases is that of tests per day per 1000 inhabitants. That could also be observed by looking at the graphs individually, the number of total tests performed per 1000 inhabitants decreases the percentage of undocumented infections at a much lower rate than the number of tests per day per 1000 inhabitants.

Both methods found a region of agreement (99.5%–99.7%) of undocumented infections in Brazil. Since both methods match closely, we decided to accept the estimate for undocumented infections in Brazil and moved on to the simulations of infection and death curves of the country and of some of its specific regions.

## 4 Simulations

For the simulation of the whole of Brazil, we used the *World Population Prospects* from the United Nations (UN) to evaluate the age distribution in Brazil in the year of 2020 (United Nations & Affairs, 2019) (This distribution will be used when simulating the expected scenario for the whole country; when considering more specific age distributions for each state, we acquired data from the Brazilian Institute of Geography and Statistics (IBGE) census, mentioned in the following sections regarding each state). We found no study measuring the social contact matrix for the country, but the study (Deps et al., 2006) evaluated the high levels of social contact in Brazil as an important factor for the spreading of leprosy. Therefore, we decided to use the social contact matrix found with the highest entries among those available (Poland) due to the Brazilian’s culture of proximity.

**Fig. 6:**
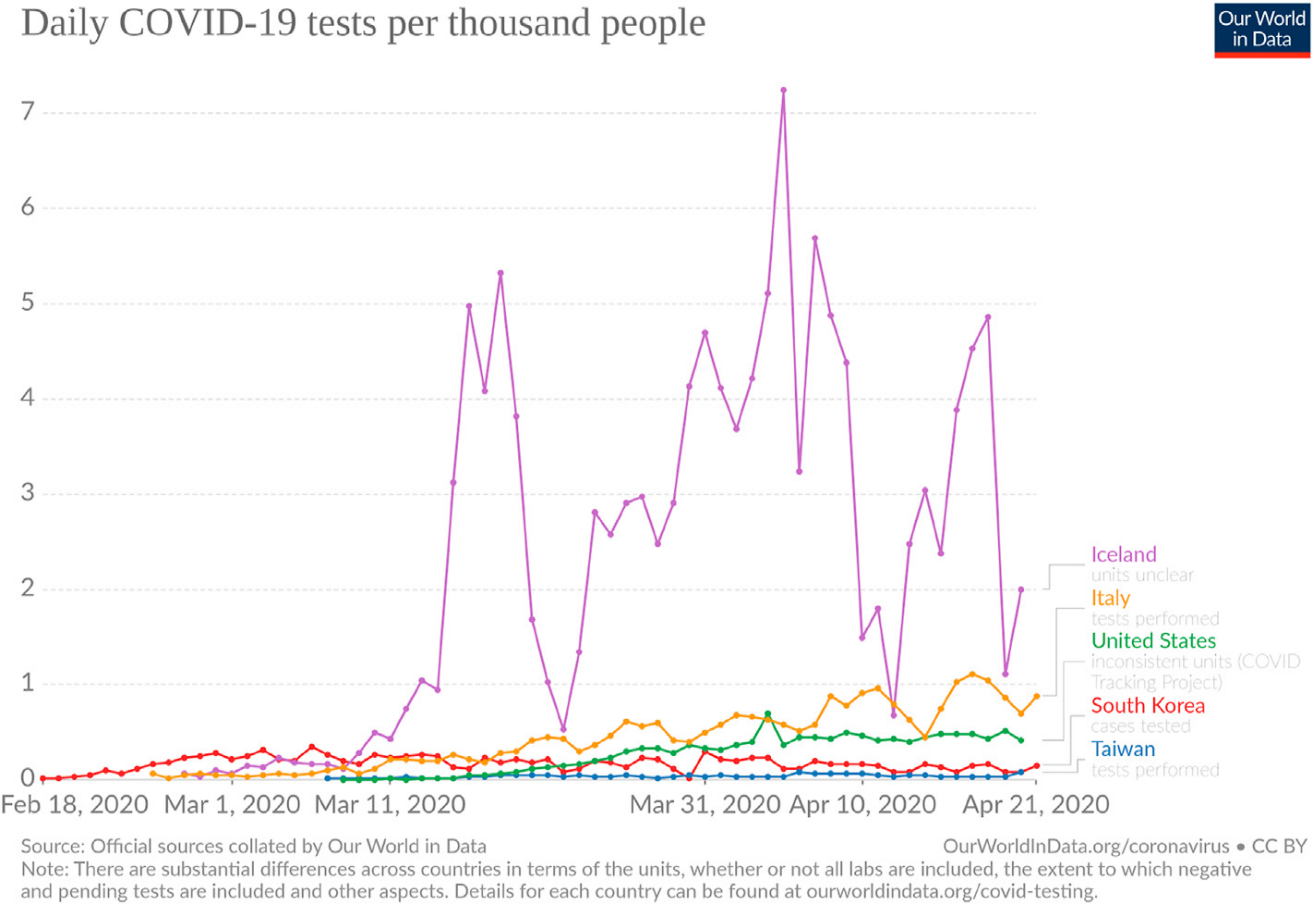
Tests per day per 1000 inhabitants. Taken from (Max Roser & Ortiz-Ospina, 2020).

**Fig. 7:**
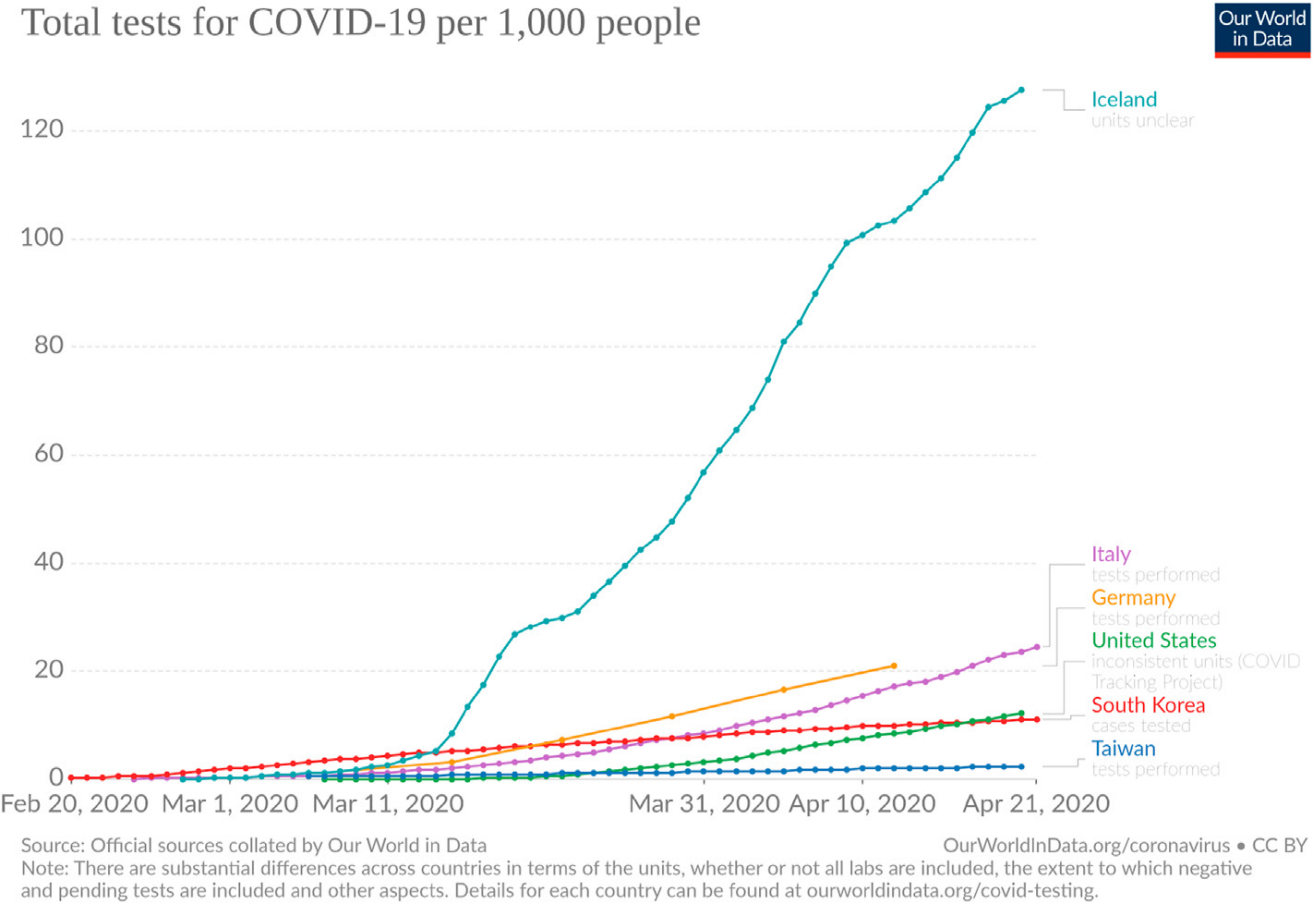
Total number of tests per 1000 inhabitants. Taken from (Max Roser & Ortiz-Ospina, 2020).

For the values of γ and μ we choose to use the ones found in the data of South Korea, Germany, Iceland and Taiwan, since these countries are performing more tests per 1000 inhabitants than Brazil, making their data more reliable (Fig. 6, Fig. 7). For each country, we acquired the average values for $\tau_{d}$ and $\tau_{r}$, knowing the CFR.

Data from Taiwan presented large fluctuations in the behavior of μ and γ, even with a almost constant Case Fatality Rate (CFR) of 1.3%±0.2%, making the values for $\tau_{d}$ and $\tau_{r}$ inconclusive. That might be explained by the early intervention made by the local government, drastically changing the values for the parameters. Clinical studies performed on Wuhan patients found $\tau_{d}$ on average 18 days (6–32) (Ruan et al., 2020), and 20 days (17–24) (Wu et al., 2020).

When fitting the data of those countries with the model to extract β (Table 1, Table 2), we took into consideration in the simulations the non-pharmaceutical intervention in each country in order to better describe β. The value of β was used to set a reference to compare with the ones found with the fitting of data from each state.

**Table 1:** Average values of $\tau_{d}$ and $\tau_{r}$ acquired from data.

Country	$\tau_{d}$	$\tau_{r}$
Iceland	14.3 ± 4.3 days	11.5 ± 4.5 days
South Korea	13.5 ± 5.6 days	21 ± 10 days
Germany	13.6 ± 5.8 days	17.5 ± 8 days
Average	13.8 ± 5.2 days	16 ± 7.5 days

**Table 2:** Values of β.

Country	β
Taiwan	0.427 ± 0.066
Germany	0.483 ± 0.025
South Korea	0.534 ± 0.040
Iceland	0.685 ± 0.121
Average	0.532 ± 0.063

For the incubation period $c^{-1}$, we took an average of the values found in previous studies (Table 3).

**Table 3:** Incubation time of the disease according to other studies with an average of 5.1 days.

incubation time	95% confidence	Reference
6.4 days	5.6–7.7	Backer et al. (2020)
5.2 days	4.1–7	Li et al. (2020b)
5 days	–	Linton et al. (2020)
4 days	–	Guan et al. (2020)
5.1 days	4.5–5.8	Lauer et al. (2020)

The value for *k* was set to 44% of β based on the findings that showed that presyntomatic cases were responsible for 44% of the infections (He et al., 2020). The parameter $P_{IFR}$ for each age group was set by re-scaling the international average of the case fatality rate (CFR) (WorldMeters, 2020) with the estimated infection fatality rate of 0.7% (Salje et al., 2020), ranging from 0.001% for those younger than 20 years old to 10.1% to those older than 80 years old (Table 4), while $P_{survival}=1-P_{IFR}$.

**Table 4:** IFR of COVID-19 for different ages.

Age (years)	Infection Fatality Rate
0–9	0.001
10–19	0.001%
20–29	0.001%
30–39	0.06%
40–49	0.12%
50–59	1.2%
60–69	2.5%
70–79	7.0%
+80	10.1%

To simulate the ICU population, we added the hospitalized population *H* to the set of differential equations (1)–(5). The introduction of this compartment is done by removing individuals from (3) with rate $P_{h}/\tau_{h}$, where $P_{h}$ is the fraction of infections that are critical and require ICU units, and $\tau_{h}$ is the average time from the symptoms onset to admission to the ICU. Inside the *H* compartment, individuals are removed to the death compartment with the rate $\mu_{h}=P_{dh}/\tau_{dh}$, where $P_{dh}$ is the probability of dying upon ICU entry and $\tau_{dh}$ is the average time from ICU admittance to death. Similarly, individuals are also removed to the recovered group with the analogous rates $\gamma_{h}=\left(1-P_{dh}\right)/\tau_{rh}$. The result is the following modification in equations (3) to (5)

(11) $$\frac{dI}{dt}=cE\left(t\right)-\left(1-P_{h}\right)\gamma I\left(t\right)-\left(1-P-h\right)\mu I\left(t\right)-\frac{P_{h}}{\tau_{h}}$$

(12) $$\frac{dH}{dt}=\frac{P_{h}}{\tau_{h}}-\gamma_{h}H\left(t\right)-\mu_{h}H\left(t\right)$$

(13) $$\frac{dR}{dt}=\gamma I\left(t\right)+\gamma_{h}H\left(t\right)$$

(14) $$\frac{dD}{dt}=\mu I\left(t\right)+\mu_{h}H\left(t\right)$$

Table 5 contains the parameters for the simulation of the ICU population.

**Table 5:** Parameters for the simulation of the ICU population.

Parameter	Value	Reference
$P_{h}$	0.015	adapted from (Salje et al., 2020)
$\tau_{h}$	3.5 days	Arentz et al. (2020)
$\tau_{rh}$	16 ± 4 days	Chen et al. (2020)
$\tau_{dh}$	7 (3–11) days	Yang et al. (2020)
$P_{dh}$	0.52	Arentz et al. (2020)

## 5 Results

In the simulation for the whole country, we considered *N* to be 5% of the total population based on an international behavior for the total number of infections in other countries (Salje et al., 2020). We also selected $P_{Inf}=14\text{\%}$ according to (Rocha Filho et al., 2020).

In order to add the effect of the use of masks by a large number of individuals to the simulation, we use a logistic function to decrease the value of $P_{infec}$ by 50% based on (MacIntyre et al., 2011), the slope of the decreasing region was set to be 10x slower than the one simulated with social distancing. We also choose $P_{d}=0.5$, taking the national average for the population in social isolation (inloco, 2020).

**Fig. 8:**
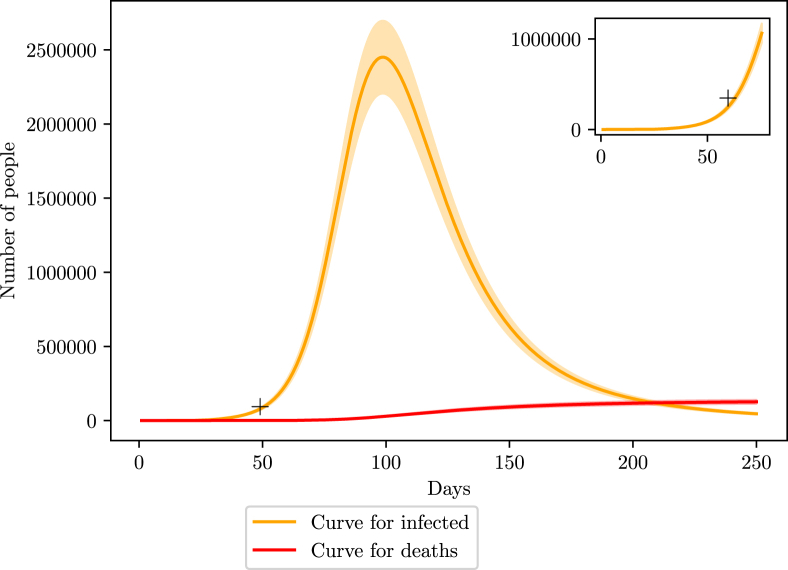
Simulation of the COVID-19 pandemic crisis in Brazil. Red curve shows the number of deaths caused by COVID-19 while orange curve represents the active number of infections. The shaded area represents the variation margin around the prediction. The smaller window on the top right corner shows an enlargement of the region close to the estimated number of infections described in section 3.

The curve (Fig. 8) shows a good agreement with the estimated values by the number of SARS hospitalizations in the last weeks of March, shown by the + mark on the graph. We also predict that the peak of the infection curve in Brazil should occur 100 days after the first case, which we considered to be at the beginning of February. Therefore, the peak should be in the middle to end of May with 2.4 million infections, ranging from 2.2 to 2.7 million. The number of deaths is estimated to be around 126 thousand, ranging from 114 to 139 thousand. By the end of the first wave, we estimate 8 million infections, ranging from 6.4 to 9.6 million.

The shaded areas represent a 10% deviation from the simulated curve. The value of the deviation was chosen as a reflection of the uncertainty in the value for the effective population *N*.

### 5.1 Pernambuco

Online data available from the local government in (CIEVSPE, 2020) states a total of 0.84 tests per 1000 inhabitants and an average of 0.05 tests per day per 1000 inhabitants, meaning that more than 90% of infections are being undocumented. For the simulation, we acquired data regarding the age and geographical distribution of the population from the last census from IBGE (IBGE, 2017a, IBGE, 2017b, IBGE, 2017c, IBGE, 2017d).

The official record for the first case dates to the 12th of March, however, data from (CIEVSPE, 2020) now shows a ICU entry of a 71 year old man in the capital of the state, Recife, diagnosed with the virus SARS-CoV-2 before this date. The patient started having symptoms on March 1st. We choose to set this date as the starting point of the simulation. According to (inloco, 2020), the isolation index, which measures the fraction of the population in social isolation is on average 50%.

**Fig. 9:**
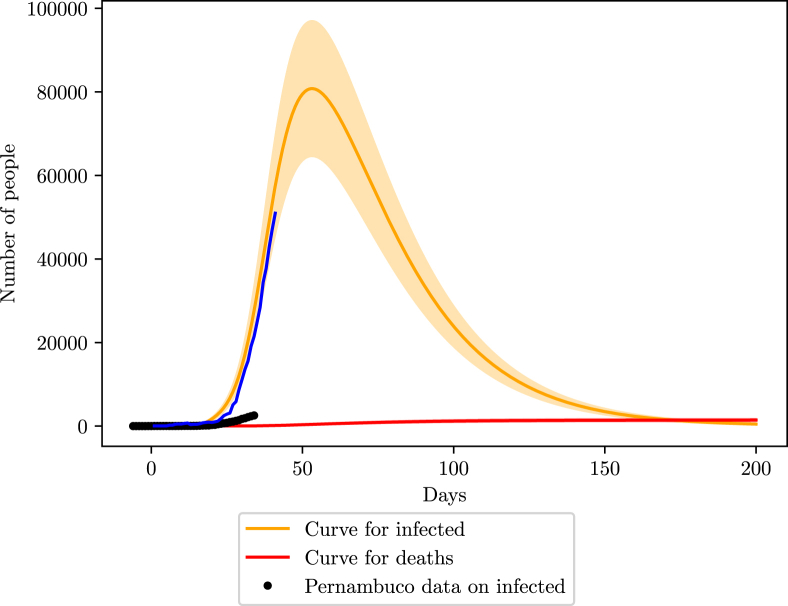
Simulation of the COVID-19 pandemic crisis in Pernambuco. The black dots are the reported number of active infections, done by subtracting the deaths and recoveries from the number of cumulative infections, the blue curve shows the behavior of the active infections data considering 90% loss of infections, that is, dividing the reported data by 0.1.

The simulation shows a peak close to the 50th day, in the beginning of May, with 8000 infections, ranging from 6000 to 10000 cases. The number of deaths estimated is 1400 (1167–1680). Here we increased the margin of error to 20%, to represent a larger uncertainty on *N* at specific locations (Fig. 9).

Despite the large number of cases lost, when fitting the data with a simulated curve, the value of β is 0.460±0.050, which agrees with the international standards. That indicates that the good tracking of the rate of change of the infection curve is good in Pernambuco. The state might not have the precise values of the real infections, but it has a good knowledge of their growth. This is an important feature for the state to be able to say that its data might represent the real scenario on a smaller scale.

The state of Pernambuco has a total of 1315 ICU beds according to a census carried by the Brazilian Association of Intensive Medicine (AMIB) in the year 2016 (A. de Medicina Intensiva Brasileira, 2016). However, recent news point to 80% of these beds already being occupied, bringing the available number of ICU beds to 263.

**Fig. 10:**
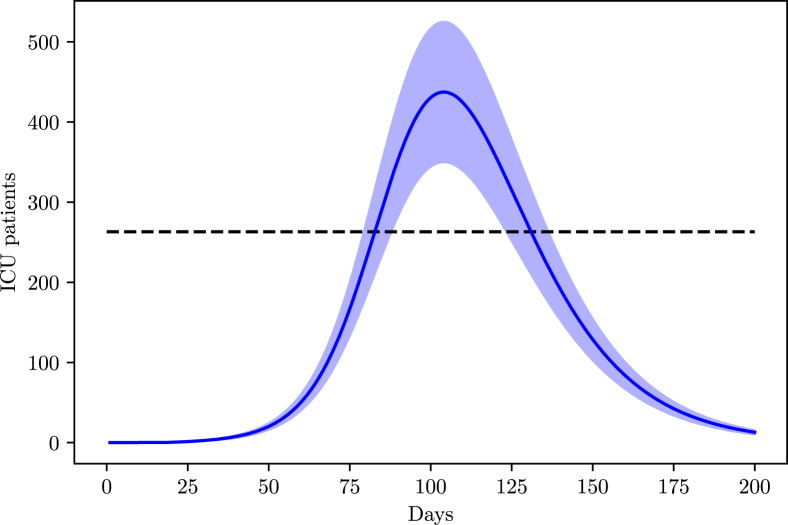
Simulation of the population in the ICU in Pernambuco due to COVID-19.

From Fig. 10 we expect a higher ICU demand than the maximum capacity for Pernambuco, however, the capacity may be increased with the construction of campaign hospitals.

### 5.2 Espirito Santo

In Espirito Santo, the online data provided by the government states a total of 6.70 tests per 1000 inhabitants realized, placing the uncertainty percentage close to 88% (78–98). There are also 161 ICU units available for COVID-19 cases (G. do Estado do Espírito Santo, 2020). The population data for the simulations was retrieved from a local census done by IBGE (IBGE, 2017a, IBGE, 2017b, IBGE, 2017c, IBGE, 2017d). The isolation index is on average 45% (inloco, 2020).

Like Pernambuco, the fitting on the Espirito Santo data reveals a good agreement of β with international parameters, β=0.436±0.199. However, the large error margin of the data lowers the confidence in it.

We found no record of previous hospitalizations due to COVID-19 prior the first case announced on March 6th, as we did for Pernambuco. Therefore, we chose the official day as the starting point of the disease. The first infectious individual was in the 30–39 years old age group.

**Fig. 11:**
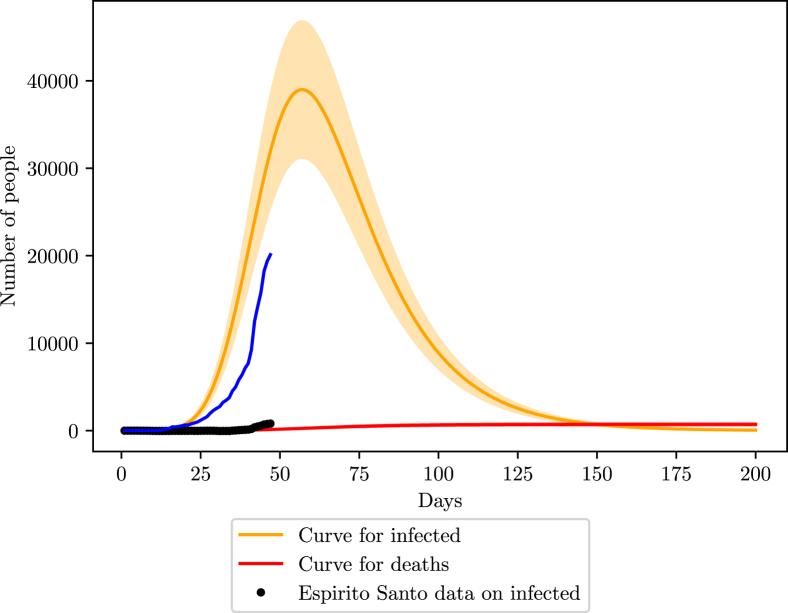
Simulation of the COVID-19 pandemic in Espirito Santo.

The peak in Espirito Santo is close to 70 days after the start, close to May 15th, with a maximum infection number of around 40000 (48000 - 32000) as shown in Fig. 11. The number of deaths is estimated to 700 (560–840).

**Fig. 12:**
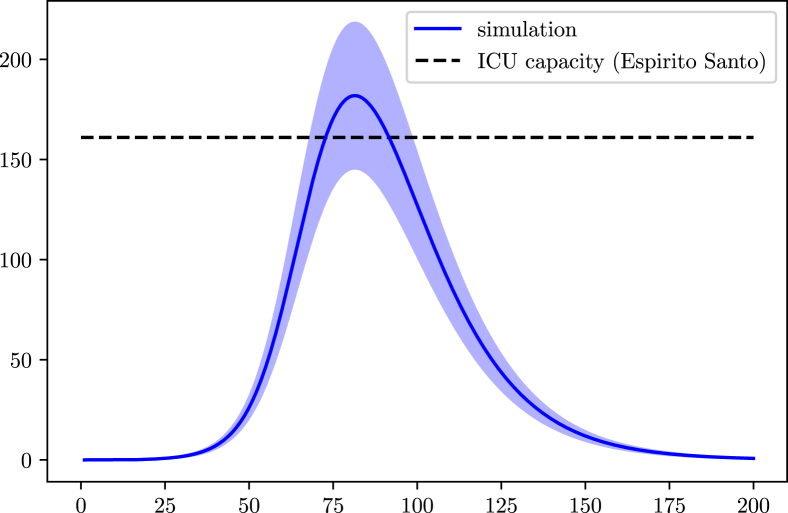
Simulation of the population in the ICU in Espirito Santo due to COVID-19. The blue curve represents the data according the 88% of undocumented infections.

Regarding the ICU demand we expect minor or no issues for the state, according to the current levels of social isolation (Fig. 12).

### 5.3 Federal District

Recent data from the government reveals 20716 tests, meaning 6.8 tests per 1000 inhabitants, meaning the state most likely has 86% (78.5–94.2) of undocumented infections. Unfortunately, no record of tests per day was found, so better accuracy on lost cases was not possible. The first registration of COVID-19 on the region is from the 5th of March, with non-pharmaceutical interventions starting on the 10th of March (S. de Saúde do Distríto Federal, 2020).

Like in the previous states, the IBGE census was used to extract the population’s distribution (IBGE, 2017a, IBGE, 2017b, IBGE, 2017c, IBGE, 2017d).

The fit of the data with the simulations returns an efficiency of 88% of social isolation, but β and $\tau_{d}$ are off the margin of acceptance, indicating that the state is not efficiently tracking the rate of the increase of deaths and cases, possibly invalidating the estimated percentage of the efficiency of the social isolation. The isolation index according to (inloco, 2020) is on average 50%.

**Fig. 13:**
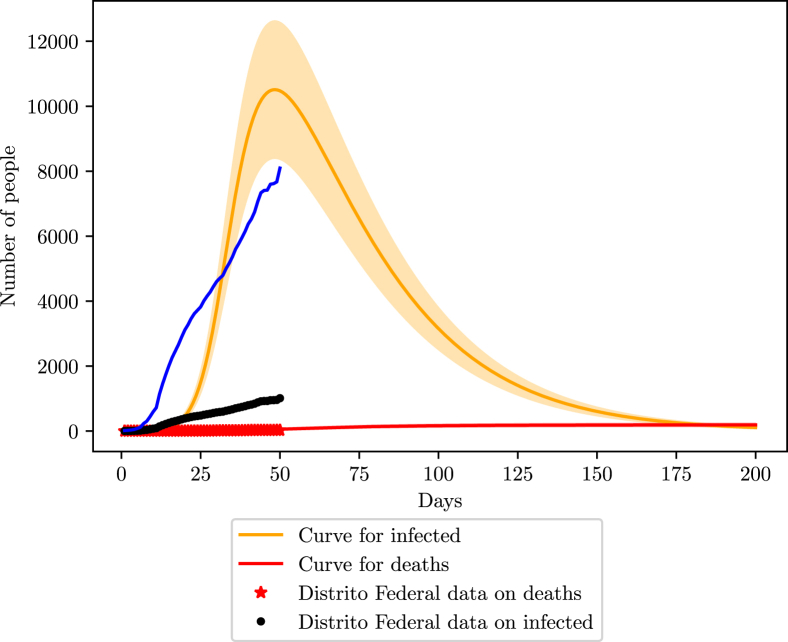
COVID-19 scenario for the Federal District. The blue curve represents the behavior of the data considering the 86% loss of infections.

The simulation shows that the Federal District is currently at its highest number of infections, around 10000 (8000–12000). The maximum number of deaths is projected to reach 190 (158–228). Also, with the current number of infections, the Federal District is losing 89% of its cases (87–91%), in agreement with the margin estimated by the number of tests performed (Fig. 13).

From the AMIB census, the state possesses 659 ICU beds. We assume an occupation of 80% before the disease reached the state.

**Fig. 14:**
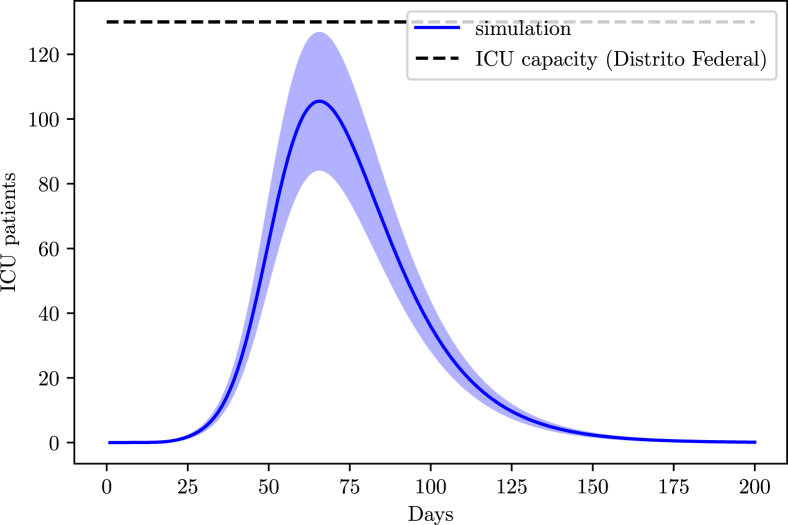
ICU demand in the Federal District due to COVID-19.

According to the simulation for the Federal District, at the current social distancing level, it is not expected to encounter hospitalization issues (Fig. 14).

### 5.4 Sao Paulo

The state of Sao Paulo also provided online data gathered by the government (G. do Estado de São Paulo, 2020). The first infection notified dates from the 26th of February. Studies done with cellphone data from Sao Paulo inhabitants saw an average of 53.6%±3.4% of the population is respecting the social isolation imposed by the local government on 24th March (G. do Estado de São Paulo, 2020).

When fitting the data with the model, considering a non-pharmaceutical intervention starting 27 days after the first case, we found an efficiency of 58.3%±7% in social isolation measures, in agreement of the study. We also found β=0.454±0.52. Unfortunately, the government did not display data on infections, but with such a high mortality rate of, around 8%, the number of infections is probably 4x bigger than the official number (meaning 75% of undocumented infections), assuming that the number of deaths is in good agreement with the real scenario. However, given the behavior of previous states, and the general scenario of Brazil, it is most likely that Sao Paulo finds itself in a 90% loss scenario.

**Fig. 15:**
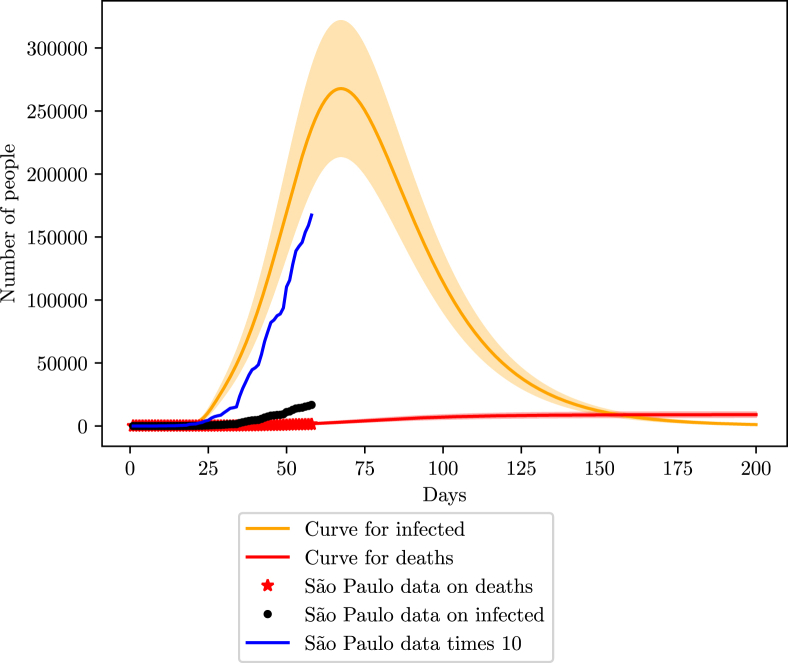
Simulation of the COVID-19 pandemic in Sao Paulo. The blue curve represents the 90% loss of data in Sao Paulo considering a constant testing rate.

**Fig. 16:**
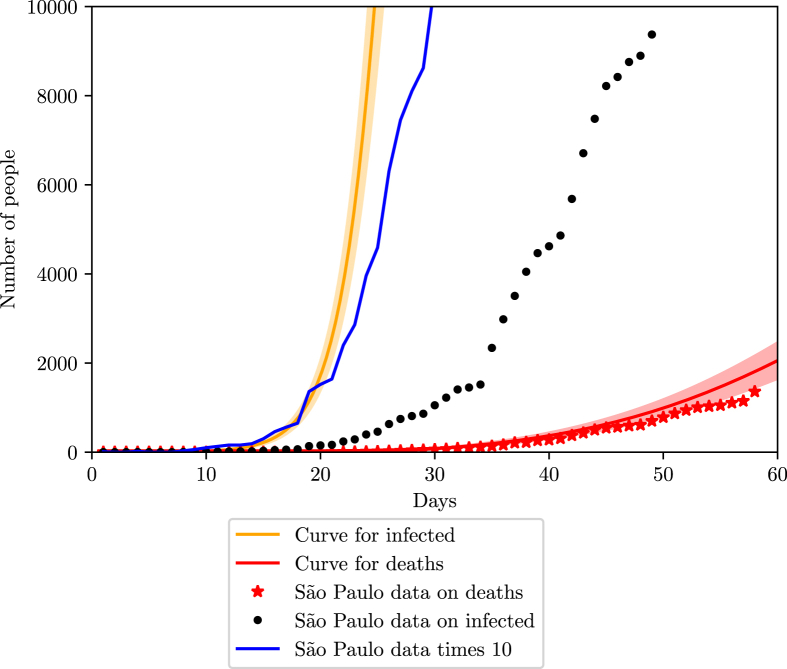
Simulation of the COVID-19 pandemic in Sao Paulo for recent data (April 23rd).

The state has its peak projected to be around the 70th day of infection, or close to the 7th of May (Fig. 15, Fig. 16). The peak number of infections should be 260000 (208000–312000). For the number of deaths, the estimate is close to 6500 (5200–7800).

From the AMIB census, the state of Sao Paulo has a total of 7312 ICU beds and recent news point to 53% of them already being occupied, leaving around 3400 ICU beds available for COVID-19 treatment.

**Fig. 17:**
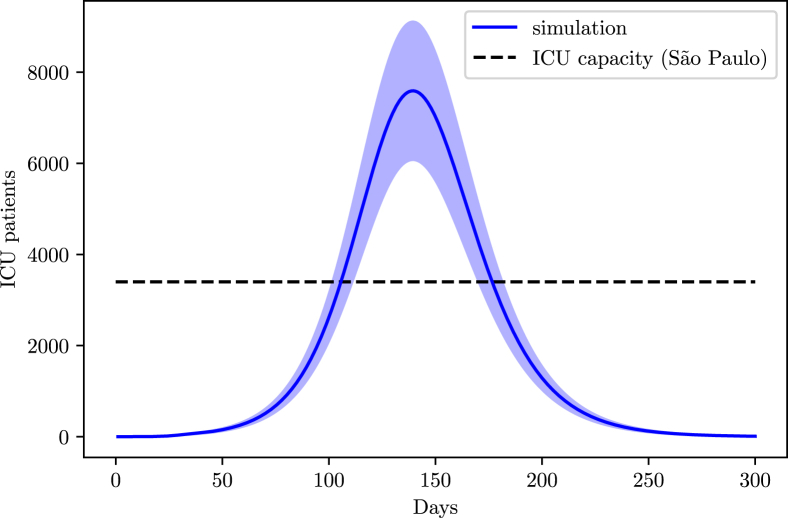
Simulation of the ICU demand in the state of Sao Paulo.

Fig. 17 predicts a long period of hospitalizations problems for the state of Sao Paulo, with a peak demand of ICU units twice as high as the current capacity.

### 5.5 Amazonas

For Amazonas, the fitting of data acquired from the Health Ministry yields β=0.406±0.096 and $\tau_{d}=16\pm 6$, showing that, despite the high number of undocumented infections, the state is in the same situation found in other states; knowing the behavior of the curve, but not the true number of each point on the curve. The difference from previous states is that the value of $\tau_{d}$ is also in agreement with international values. The average isolation index for Amazonas is around 51%.

The census from IBGE (IBGE, 2017a, IBGE, 2017b, IBGE, 2017c, IBGE, 2017d) was also used here to acquire population data for the state.

From the AMIB census, Amazonas possesses 249 ICU beds, with 55% of them being occupied before the outbreak. Unfortunately, no data on tests was found for Amazonas, therefore we consider a 90% loss of infections.

**Fig. 18:**
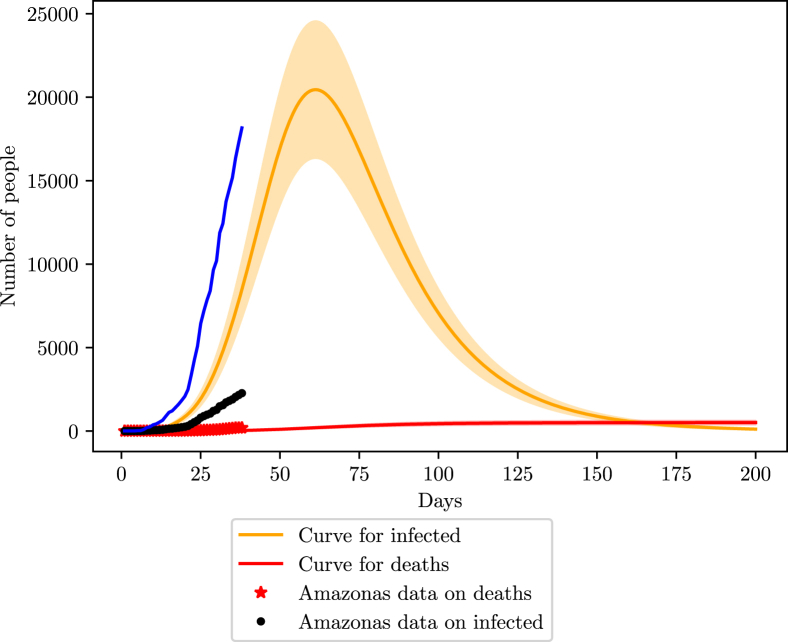
Simulation of the COVID-19 pandemic in Amazonas.

**Fig. 19:**
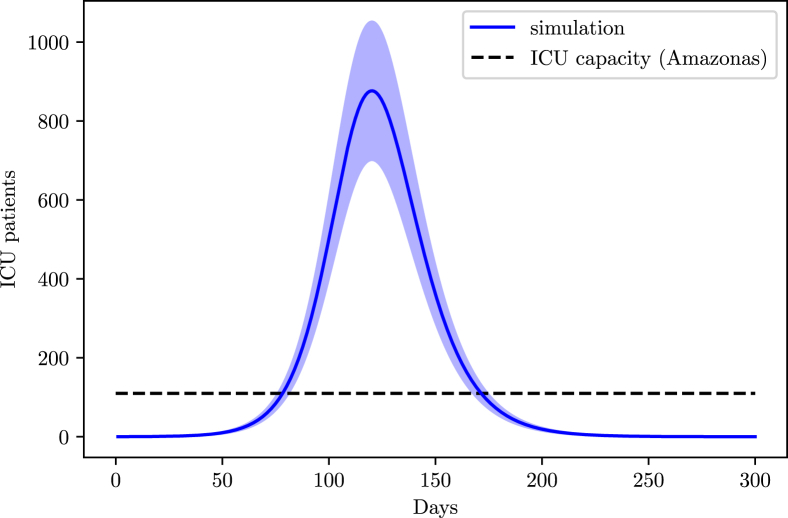
Simulation of the ICU demand in the state of Amazonas.

Amazonas peak is estimated to occur on May 16th, with a 20000 infections peak (16000–24000). Deaths are estimated to reach 500 in total (400–600) (Fig. 18). Several hospitalization issues are expected during the pandemic through the region (Fig. 19); given the concentration of indigenous tribes throughout the Amazon rain forest territory, it should be expected for there to be a high density of cases in the indigenous population.

## 6 Future scenarios

Simulating the halting of non-pharmaceutical interventions is equivalent to making β increase up to it’s initial value. By making such simulations, we observe an increase of cases, that is, a second peak of the disease right after the halt.

**Fig. 20:**
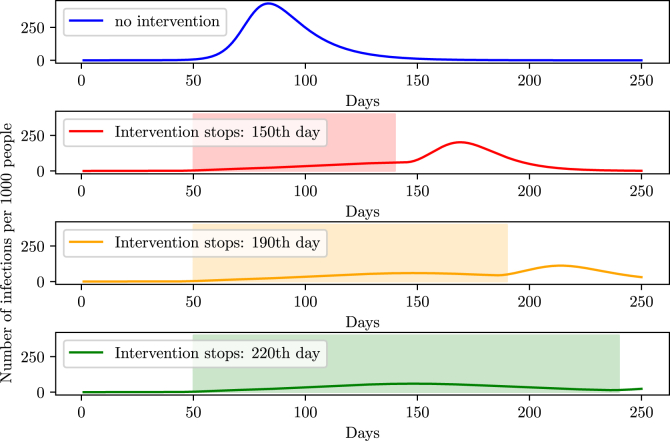
Height of the second peak for different times at which social isolation is halted.

Fig. 20 shows that to drastically diminish the second peak, the social isolation must endure about 220 days supposing an efficiency of 70%. It is equivalent to stating that in Brazil, quarantine should hold out until October, while for a total prevention of the second peak, social isolation must take place until December. That is expected and agrees to other simulations made by another group from the University of Harvard which projected that, to prevent a second peak in the world and the possible re-incidence of the virus, social isolation must hold until the beginning of 2021 and social distancing until 2022 or 2024 (Kissler et al., 2020).

**Fig. 21:**
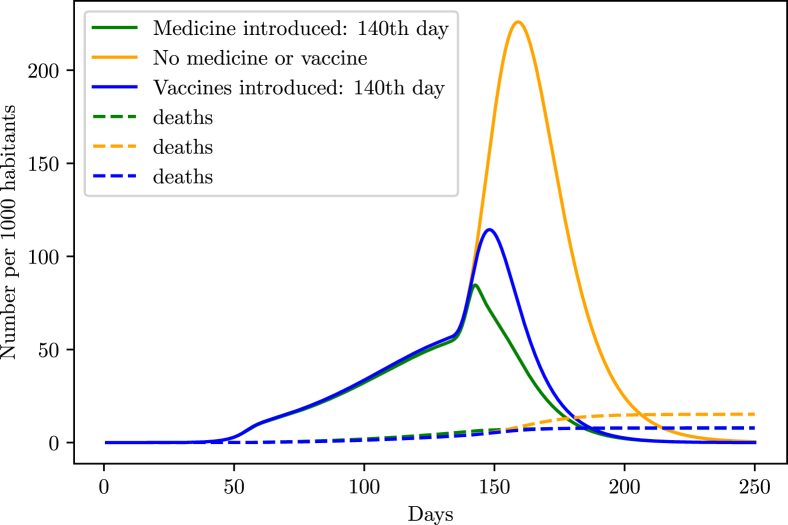
Behavior of the infection curve if the vaccination/medication occurs at the same time of intervention stopping.

**Fig. 22:**
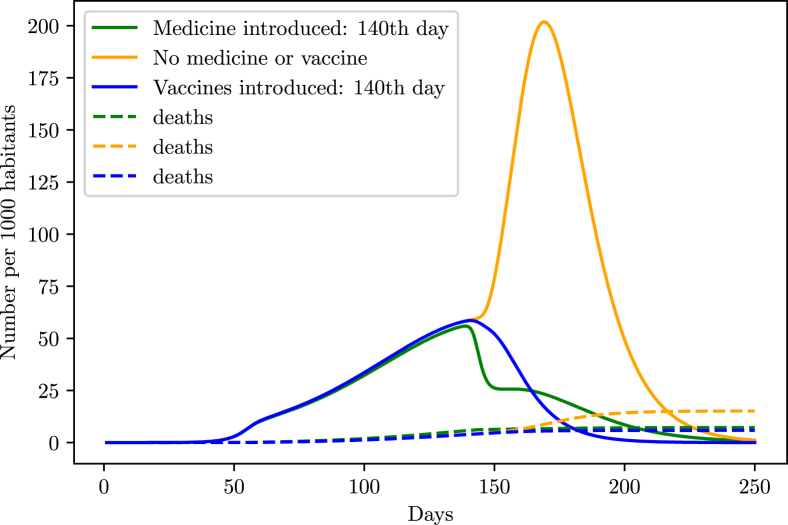
Behavior of the infection curve if the vaccination/medication occurs 10 days before halting the intervention.

However, that scenario might drastically change with the introduction of vaccines or efficient medicine in the population. As shown in the simulations (Fig. 21, Fig. 22), such pharmaceutical interventions are able to rapidly decrease the infection curve. In order to simulate the effect of medicine in the population, we started decreasing the death probability $P_{IFR}$ and time taken from the symptoms onset to the recovery $\tau_{r}$ from a specific date, until it reaches a maximum value. We supposed that the introduction of medicine decreased both $P_{IFR}$ and $\tau_{r}$ by half in the period of 10 days after the introduction in the population.

For the vaccines, we added the term −vS(t) in (1), which takes out individuals from the susceptible group at a rate *v* called vaccination rate, and added the term vS(t) in (4), adding those individuals on the recovery group, granting them immunity against the virus. The vaccination rate *v* was chosen to behave according to a logistic function starting at t=0, and gradually increasing to 0.2 after a specific time.

From the simulations, the safest method is not to stop the intervention and introduce the vaccines or drugs into the population, and then wait a small period of 10 days before halting the intervention.

## 7 Discussion with other studies

Other studies performed for Brazil found interesting results regarding the action of intervention policies, Bastos and Daniel simulated an epidemic scenario for Brazil with social isolation and found that if social isolation does not last long enough, the effect of decreasing the infection curve is instead substituted by a shift in the peak of the infection curve (Bastos & Cajueiro, 2003, p. 14288). Furthermore, previous models considering the implementation of public policies of social isolation have managed to show a direct relation of the reduction of daily infections in Brazil to social isolation (Crokidakis, 2020).

Simpler simulations using a different compartmental model suggest a possible infection peak of $10^{8}$ cases in Brazil (Crokidakis, 2020), however they do not consider the introduction of the incubation period, causing a higher growth rate for the disease (Cintra et al., 2020), which in turn causes a tendency of predicting the infection peak sooner than expected.

Our work proposes not only expected scenarios, but includes an evaluation of undernotified cases and ICU demand. Finally, Brazil being a tropical country means changes in temperature affect the spread of the virus throughout the national territory (Prata et al., 2020). Future research could be performed combining the average temperature during the pandemic to better forecast viral spread.

## 8 Conclusion

Simulations of the COVID-19 outbreak vary from model to model, here we try to find balance between the most precise model, which could be achieved considering also a group of asymptomatic infections, and the availability of data. With this objective, we decided to simulate the behavior of the disease in Brazil based on international parameters under the assumption that they would not differ much from Brazil, for example the average time from the onset of symptoms to hospitalization found in Shanghai, and the main aspects regarding the transmission would be intervention policies, population demographics and social contact. This assumption might prove to be limited if is later found that climate effects strongly alters the spread, since Brazil is a tropical country with a higher average temperature when compared to Europe and Asia, where many parameters of the simulations were found.

Another limitation of the model is in the assumption of a homogeneous population. We tried here to counter act this limitation by estimating the effective population *N* according to international parameters and by widening the error margin of the predictions. A better estimate of the outbreak could be done by assessing cities individually, however that would represent a loss of data, since demographics available by IBGE are mainly on states and major cities. Another outtake would be the testing data, the states which provided testing data, did only for the whole state but not for individual cities. We did not consider comorbidities in the population such as diabetes and cancer, however the age of the individual seems to be the most important factor in determining mortality factors (N. U. Kingdom, 2020).

We also state here that the nature of the process is stochastic, allowing fluctuations from the deterministic model used to run the simulations. Thus, this study present an estimate of the real situation and expected behavior given the parameters associated with the disease and the efficiency of the intervention. The above results present the dimension of the real scenario, but due to possible initial fluctuations in the stochastic behavior in reality, we might find some deviations from the expected.

Even with limitations, the model has proven efficient in generating curves that agree with the estimated loss of cases for each state. From the states studied here, Sao Paulo, Amazonas and Pernambuco present the highest risk of collapse in the health system, while Espirito Santo and the Federal District should have minor issues with system collapse or none at all. The blue curve representing the behavior of the official data considering the error percentage for Amazonas exhibited a growth far from the simulation region, however, it falls perfectly inside this region when data is translated by 10 days, meaning that if the infection in Amazonas begun 10 days earlier than previously thought, the data fits the simulated curve.

In the case of the duration of social isolation, the safer situation is to hold the isolation for as long as possible in order to decrease the second peak height, while increasing the number of tests performed. All simulations considered here did not assume the end of the intervention, therefore, numbers of deaths may be higher. Should any efficient drugs in combating the virus come along, the simulations show the safer way is to first introduce them in the population without breaking the social isolation, and about 10 days later start the process of reopening.

## SUPPLEMENTARY MATERIAL

Source code used for some simulations and with didatic example of predictions available at https://github.com/PedroHPCintra/Coronavirus.

## References

[bib1] inloco (2020). https://mapabrasileirodacovid.inloco.com.br/pt/.

[bib2] A. de Medicina Intensiva Brasileira (2016). Censo amib.

[bib3] Andersen K.G., Rambaut A., Lipkin W.I., Holmes E.C., Garry R.F. (2020). The proximal origin of sars-cov-2. Nature Medicine.

[bib4] Arentz M., Yim E., Klaff L., Lokhandwala S., Riedo F.X., Chong M., Lee M. (2020). Characteristics and outcomes of 21 critically ill patients with covid-19 in Washington state. JAMA.

[bib5] Backer J.A., Klinkenberg D., Wallinga J. (2020). Incubation period of 2019 novel coronavirus (2019-ncov) infections among travellers from wuhan, China, 20–28 january 2020. Euro Surveillance.

[bib6] Bastos S.B., Cajueiro D.O. (2003). Modeling and forecasting the covid-19 pandemic in Brazil.

[bib7] Chen J., Qi T., Liu L., Ling Y., Qian Z., Li T., Xu S. (2020). Clinical progression of patients with covid-19 in shanghai, China. Journal of Infection.

[bib8] CIEVSPE (2020). http://dados.seplag.pe.gov.br/apps/corona.html.

[bib9] Cintra P., Citeli M., Fontinele F. (2020). Mathematical models for describing and predicting the covid-19 pandemic crisis.

[bib10] COVID C., Team R. (2020). Severe outcomes among patients with coronavirus disease 2019 (covid-19)––United States, february 12–march 16, 2020. MMWR Morb Mortal Wkly Rep.

[bib11] Crokidakis N. (2020). Covid-19 spreading in rio de janeiro, Brazil: Do the policies of social isolation really work?. Chaos, Solitons & Fractals.

[bib12] Crokidakis N. (2020). ‘‘Modeling the early evolution of the covid-19 in Brazil: Results from a susceptible-infectious-quarantined-recovered (siqr) model. International Journal of Modern Physics C.

[bib13] Cui J., Li F., Shi Z.-L. (2019). Origin and evolution of pathogenic coronaviruses. Nature Reviews Microbiology.

[bib14] Deps P.D., Guedes B., Bucker Filho J., Andreatta M.K., Marcari R.S., Rodrigues L.C. (2006). Characteristics of known leprosy contact in a high endemic area in Brazil. Leprosy Review.

[bib15] Ferguson N., Laydon D., Nedjati Gilani G., Imai N., Ainslie K., Baguelin M., Cuomo-Dannenburg G. (2020). Report 9: Impact of non-pharmaceutical interventions (npis) to reduce covid19 mortality and healthcare demand.

[bib16] Flaxman S., Mishra S., Gandy A., Unwin H., Coupland H., Mellan T., Perez Guzman P. (2020). Report 13: Estimating the number of infections and the impact of non-pharmaceutical interventions on covid-19 in 11 european countries.

[bib17] G. do Estado do Espírito Santo (2020). https://coronavirus.es.gov.br/painel-covid-19-es.

[bib18] G. do Estado de São Paulo (2020). https://www.saopaulo.sp.gov.br/coronavirus/.

[bib19] Guan W.-j., Ni Z.-y., Hu Y., Liang W.-h., Ou C.-q., He J.-x., Hui D.S. (2020). Clinical characteristics of coronavirus disease 2019 in China. New England Journal of Medicine.

[bib20] He X., Lau E.H., Wu P., Deng X., Wang J., Hao X., Tan X. (2020). Temporal dynamics in viral shedding and transmissibility of covid-19. Nature Medicine.

[bib21] IBGE (2017). https://cidades.ibge.gov.br/brasil/pe/panorama.

[bib22] IBGE (2017). https://cidades.ibge.gov.br/brasil/es/panorama.

[bib23] IBGE (2017). https://cidades.ibge.gov.br/brasil/df/panorama.

[bib24] IBGE (2017). https://cidades.ibge.gov.br/brasil/am/panorama.

[bib25] Kissler S.M., Tedijanto C., Goldstein E., Grad Y.H., Lipsitch M. (2020). Projecting the transmission dynamics of sars-cov-2 through the postpandemic period. Science.

[bib26] Lauer S.A., Grantz K.H., Bi Q., Jones F.K., Zheng Q., Meredith H.R., Lessler J. (2020). The incubation period of coronavirus disease 2019 (covid-19) from publicly reported confirmed cases: Estimation and application. Annals of Internal Medicine.

[bib29] Li R., Pei S., Chen B., Song Y., Zhang T., Yang W., Shaman J. (2020). Substantial undocumented infection facilitates the rapid dissemination of novel coronavirus (sars-cov2). Science.

[bib28] Linton N.M., Kobayashi T., Yang Y., Hayashi K., Akhmetzhanov A.R., Jung S.-m., Yuan B., Kinoshita R., Nishiura H. (2020). incubation period and other epidemiological characteristics of 2019 novel coronavirus infections with right truncation: A statistical analysis of publicly available case data. Journal of Clinical Medicine.

[bib27] Li Q., Guan X., Wu P., Wang X., Zhou L., Tong Y., Wong J.Y. (2020). Early transmission dynamics in wuhan, China, of novel coronavirus–infected pneumonia. New England Journal of Medicine.

[bib30] Liu Y., Gayle A.A., Wilder-Smith A., Rocklöv J. (2020). The reproductive number of covid-19 is higher compared to sars coronavirus. Journal of Travel Medicine.

[bib31] MacIntyre C.R., Wang Q., Cauchemez S., Seale H., Dwyer D.E., Yang P., Zhang Y. (2011). A cluster randomized clinical trial comparing fit-tested and non-fit-tested n95 respirators to medical masks to prevent respiratory virus infection in health care workers. Influenza and other respiratory viruses.

[bib32] Marra M.A., Jones S.J., Astell C.R., Holt R.A., Brooks-Wilson A., Butterfield Y.S., Chan S.Y. (2003). The genome sequence of the sars-associated coronavirus. Science.

[bib33] Max Roser H.R., Ortiz-Ospina E. (2020). https://ourworldindata.org/coronavirus.

[bib34] McCreary E.K., Pogue J.M. (2020). Coronavirus disease 2019 treatment: A review of early and emerging options.

[bib35] Negri E.M., Piloto B., Morinaga L.K., Jardim C.V.P., Lamy S.A.E.-D., Ferreira M.A., D’Amico E.A., Deheinzelin D. (2020). Heparin therapy improving hypoxia in covid-19 patients-a case series.

[bib36] N. U. Kingdom (2020). https://www.covid19survivalcalculator.com/research.

[bib38] Prata D.N., Rodrigues W., Bermejo P.H. (2020). Temperature significantly changes covid-19 transmission in (sub) tropical cities of Brazil. The Science of the Total Environment.

[bib39] Read J.M., Bridgen J.R., Cummings D.A., Ho A., Jewell C.P. (2020). Novel coronavirus 2019-ncov: Early estimation of epidemiological parameters and epidemic predictions.

[bib40] Rocha Filho T.M., dos Santos F.S.G., Gomes V.B., Rocha T.A., Croda J.H., Ramalho W.M., Araujo W.N. (2020). Expected impact of covid-19 outbreak in a major metropolitan area in Brazil. medRxiv.

[bib41] Ruan Q., Yang K., Wang W., Jiang L., Song J. (2020). Clinical predictors of mortality due to covid-19 based on an analysis of data of 150 patients from wuhan, China. Intensive Care Medicine.

[bib42] Salje H., Kiem C.T., Lefrancq N., Courtejoie N., Bosetti P., Paireau J., Dubost C.-L. (2020).

[bib43] S. de Saúde do Distríto Federal (2020). http://www.saude.df.gov.br/.

[bib44] Tang N., Bai H., Chen X., Gong J., Li D., Sun Z. (2020). Anticoagulant treatment is associated with decreased mortality in severe coronavirus disease 2019 patients with coagulopathy. Journal of Thrombosis and Haemostasis.

[bib45] United Nations D.o.E., Affairs S. (2019). World population prospects 2019. Highlights.

[bib37] WHO (2020).

[bib46] WorldMeters (2020). https://www.worldometers.info/coronavirus/coronavirus-cases/.

[bib47] Wu J.T., Leung K., Bushman M., Kishore N., Niehus R., de Salazar P.M., Cowling B.J., Lipsitch M., Leung G.M. (2020). Estimating clinical severity of covid-19 from the transmission dynamics in wuhan, China. Nature Medicine.

[bib48] Wu Z., McGoogan J.M. (2020). Characteristics of and important lessons from the coronavirus disease 2019 (covid-19) outbreak in China: Summary of a report of 72 314 cases from the Chinese center for disease control and prevention. JAMA.

[bib49] Yang X., Yu Y., Xu J., Shu H., Liu H., Wu Y., Yu T. (2020). Clinical course and outcomes of critically ill patients with sars-cov-2 pneumonia in wuhan, China: A single-centered, retrospective, observational study. The Lancet Respiratory Medicine.

[bib50] Zhang S., Diao M., Yu W., Pei L., Lin Z., Chen D. (2020). Estimation of the reproductive number of novel coronavirus (covid-19) and the probable outbreak size on the diamond princess cruise ship: A data-driven analysis. International Journal of Infectious Diseases.

[bib51] Zhao S., Lin Q., Ran J., Musa S.S., Yang G., Wang W., He D. (2020). Preliminary estimation of the basic reproduction number of novel coronavirus (2019-ncov) in China, from 2019 to 2020: A data-driven analysis in the early phase of the outbreak. International journal of Infectious Diseases.

